# Establishment and Characterization of MUi027-A: A Novel Patient-Derived Cell Line of Polycystic Kidney Disease with *PKD1* Mutation

**DOI:** 10.3390/jpm12050766

**Published:** 2022-05-09

**Authors:** Aung Khine Linn, Warun Maneepitasut, Alisa Tubsuwan, Narisorn Kitiyanant, Bunyong Phakdeekitcharoen, Suparerk Borwornpinyo, Suradej Hongeng, Phetcharat Phanthong

**Affiliations:** 1 Excellent Center for Drug Discovery, Faculty of Science, Mahidol University, Bangkok 10400, Thailand; agkhinelinn@protonmail.com (A.K.L.); bsuparerk@gmail.com (S.B.); 2 Molecular Medicine Program, Multidisciplinary Unit, Faculty of Science, Mahidol University, Bangkok 10400, Thailand; warun.man@mahidol.ac.th; 3 Research Department, Faculty of Medicine Siriraj Hospital, Mahidol University, Bangkok 10700, Thailand; 4 Medical Biosciences Cluster, Institute of Molecular Biosciences, Mahidol University, Nakhon Pathom 73170, Thailand; alisa.tub@mahidol.ac.th (A.T.); narisorn.kit@mahidol.ac.th (N.K.); 5 Division of Nephrology, Department of Medicine, Faculty of Medicine, Ramathibodi Hospital, Mahidol University, Bangkok 10400, Thailand; bunyong.pha@mahidol.ac.th; 6 Department of Biotechnology, Faculty of Science, Mahidol University, Bangkok 10400, Thailand; 7 Department of Pediatrics, Faculty of Medicine, Ramathibodi Hospital, Mahidol University, Bangkok 10400, Thailand; 8 Department of Anatomy, Faculty of Science, Mahidol University, Bangkok 10400, Thailand

**Keywords:** autosomal dominant polycystic kidney disease, *PKD*, patient-derived cell line, human induced pluripotent stem cells, episomal reprogramming, disease model

## Abstract

Autosomal dominant polycystic kidney disease (ADPKD) is one of the most prevalent genetic diseases affecting the kidneys. A genetically specific mutation model is required to comprehend its pathophysiology and to develop a drug treatment. In this study, we successfully developed human induced pluripotent stem cells (hiPSCs) named MUi027-A from skin fibroblasts of a patient diagnosed with ADPKD and carrying the *PKD1* frameshift mutation (c.7946_7947delCT). MUi027-A cells showed the same genetic fingerprints as the parental cells, including the presence of the *PKD1* mutation. MUi027-A hiPSCs displayed embryonic stem cell-like characteristics with the capability of differentiating into the three germ layers. Upon directed differentiation, MUi027-A hiPSCs could be differentiated into tubular organoids with the expression of renal cell markers. Furthermore, we compared the efficiency of cyst formation in two human iPSC lines with different *PKD1* mutations. When cyst formation was induced by either forskolin or blebbistatin, MUi027-A hiPSC-derived kidney organoids displayed higher frequencies of cyst formation when compared to organoids generated from an iPSC cell line with non-truncating *PKD1* mutation genotype (c.5878C > T), suggesting the presence of physiological differences in the mechanism of cyst formation between different *PKD1* mutants. Overall, we generated and characterized a novel human iPSC line with a specific *PKD* mutation and demonstrated its potential as a disease model to study the pathophysiology of genetic determinants in the development of ADPKD disease.

## 1. Introduction

Autosomal dominant polycystic kidney disease (ADPKD) is a type of polycystic kidney disease characterized by the formation of fluid-filled cysts in the kidneys [1]. Although the formation of cysts in the renal parenchyma is the most prominent feature of ADPKD, it is a systemic disorder that can affect organs in the gastrointestinal, cardiovascular, and musculoskeletal systems [2,3]. Inherited monogenic mutations in polycystin 1 (PC 1) or polycystin 2 (PC2), encoded by *PKD1* or *PKD2* genes, respectively, are the most common causes of ADPKD [4]. The majority (~80%) of ADPKD diseases are caused by mutations in *PKD1* on chromosome 16p13.3, while the rest (~15%) are attributed to mutations in *PKD2* (chromosome 4q22.1) [5]. However, both proteins are found to be co-localized and functionally co-dependent [6,7]. Polycystins are membrane proteins located in the primary cilium that mediate Wnt/Ca2+ signaling pathways. Mutations in polycystins could lead to alterations in the downstream signaling pathways, eventually resulting in the formation of cystic kidneys [3]. Although a genetic determinant for the development of a cystic kidney is well demonstrated, the presence of strong intrafamilial variations in disease development and clinical outcome suggests that the underlying molecular pathogenesis could involve genetic as well as environmental manifestations. A two-hit hypothesis suggests that cyst formation is triggered by somatic inactivation of functionally intact *PKD* in addition to germline mutation in either the *PKD1* or *PKD2* gene [8]. The mean age of onset also indicates that individuals with *PKD1* mutations are likely to develop renal failure by the age of 70 years, whereas those with *PKD2* mutations display adequate renal function at a similar age [9]. This implies the possibility of therapeutic intervention during the early onset to modulate the disease progression. A better understanding of the pathophysiology of ADPKD would benefit the development of novel treatments as well as early diagnosis of the disease.

Induced pluripotent stem cells (iPSCs) are an emerging approach to study the molecular pathogenesis of genetic diseases, as these patient-derived cells harbor the mutant gene of interest as well as other potential modifier genes within the specific genetic background [10]. Additionally, the genetic background of the isogenic iPSC line can be modulated using genome editing techniques, such as CRISPR/Cas9, to identify the potential pathways associated with the gene of interest [11]. Moreover, iPSCs could be differentiated into 2D or 3D organoid cultures and serve as a screening platform for drug discovery and disease modeling [12]. Human iPSC-derived organoids also reportedly display pathophysiological profiles that resemble in vivo structures such as insulin-producing pancreatic β cells [13], light-responsive photoreceptor cells [14], and even a functional human liver [15]. The hiPSCs are also used to model mechanisms of diabetic vasculopathy [16], Parkinson’s disease [17], long-QT syndrome [18], and other genetic disorders [19]. Although ADPKD is one of the most common life-threatening genetic diseases, only a limited number of iPSC lines with the ADPKD genotype are listed in the publicly available Human Pluripotent Stem Cell Registry (https://hpscreg.eu/ (accessed on 30 March 2022)). Therefore, the development of additional ADPKD patient-derived iPSC lines will greatly benefit in elucidating disease mechanisms and identifying new therapeutic targets.

In this study, we successfully established an iPSC cell line (MUi027-A) from skin fibroblasts of a female ADPKD patient who had the *PKD1* frameshift mutation (c.7946_7947delCT). The developed cell line maintained normal karyotype and displayed embryonic stem cell-like characteristics with consistent pluripotency marker expressions and the capability of differentiating into the three germ layers. Furthermore, significant cyst formation can also be induced with the addition of blebbistatin, a specific inhibitor of non-muscle myosin II, or forskolin, a cell-permeable diterpenoid with adenylyl cyclase activating properties, suggesting the role of this cell line as a model for studying ADPKD disease pathophysiology.

## 2. Materials and Methods

### 2.1. Fibroblast Culture, Reprogramming and Maintenance

Fibroblasts were isolated from a 42-year-old female ADPKD patient who had the *PKD1* frameshift mutation (c.7946_7947delCT) and cultured in DMEM supplemented with 10% FBS (Hyclone, Logan, UT, USA). Episomal vectors (Addgene #27077, #27078, #27080) were electroporated into fibroblasts (2 × 10^5^) using a NEON microporator (Invitrogen, Waltham, MA, USA) with 3 pulses at 1650 V for 10 min. Electroporated cells were cultured on a 6-well plate precoated with 8.7 µg/cm^2^ Matrigel (Corning, Corning, NY, USA) for 3 days. On day 4, the media was switched to E8 medium (without TGF-β) for 2 more weeks. On week 4 of post electroporation, iPSC colonies were picked and cultured in a complete E8 medium containing DMEM/F12, L-ascorbic acid-2-phosphate magnesium (64 mg/L), FGF2 (100 µg/L), TGFβ1 (2 µg/L), insulin (19.4 mg/L), sodium selenium (14 µg/L), and transferrin (10.7 mg/L). Cells were routinely passaged and stocks were kept. For passaging, cells were treated with 0.5 mM EDTA for 6–8 min and iPSC clumps were gently collected and plated onto Matrigel-coated plates at a split ratio of 1:4. Rho-kinase inhibitor (Y-27632, Sigma, St. Louis, MO, USA) as an additive was found to be critical for cell survival following thawing or sub-culture. iPSCs were maintained in a CO_2_ incubator at 37 °C with 5% CO_2_.

### 2.2. Sequencing and Short Tandem Repeat (STR) Analysis

Genomic DNA was isolated from iPSCs using Gentra Puregene Cell Kit (Qiagen, Hilden, Germany). The regions at exon 21 of the *PKD1* gene were amplified and the DNA sequence was analyzed by Molecular Genetics Laboratory at Siriraj Hospital, Thailand. STR analysis was carried out by AmpFlSTR Identifiler PCR Amplification Kit (Applied Biosystems, Waltham, MA, USA) with 16 markers (Amelogenin, D8S1179, D21S11, D7S820, CSF1PO, D3S1358, TH01, D13S317, D16S539, D2S1338, D19S433, vWA, TPOX, D18S51, D5S818, and FGA), and analyzed on a 3500/3500xL Genetic Analyzer using GeneMapper (Applied Biosystems) by Forensic Medicine Unit, Siriraj Hospital.

### 2.3. Karyotyping

MUi027-A iPSCs were seeded on a Matrigel-coated 6-well plate and allowed to grow until they reached 80% confluence. Cells were treated with 10 μg/mL KaryoMAX colcemid (Gibco) for 2 h before being harvested by incubation with 0.25% trypsin-EDTA (Gibco, Waltham, MA, USA). The cells pellet was washed once with PBS (HyClone, UT, USA) and incubated with 0.56% KCL for 15 min at 37 °C, then the cell pellet was resuspended in a fixative solution (3:1 *v*/*v* methanol/glacial acetic acid). The fixed cells were dropped onto a glass slide and air-dried. Karyotyping was performed on G-banded metaphase chromosomes using standard cytogenetic procedures. The result was examined and analyzed by Genetic Laboratory, Institute of Molecular Biosciences, Mahidol University.

### 2.4. Analysis of Pluripotency Marker Expression and Reprogramming Plasmid Integration

RNA was isolated from iPSCs using RNeasy Plus Mini Kit (Qiagen) and was converted to cDNA using RevertAid First Strand cDNA Synthesis Kit (Thermo Scientific, Waltham, MA, USA). qPCR analysis of pluripotency markers was performed using TaqMan probes (Applied Biosystems, Waltham, MA, USA) following the manufacturer’s instruction with the StepOnePlus system. The relative expression levels were calculated using the ΔCt method. Statistical significance was considered at ** *p* < 0.01. For analysis of reprogramming plasmid integration, PCR was performed on genomic DNA extracted from MUi027-A cells using primers specific to the episomal plasmids. The parental fibroblasts and a mixture of episomal plasmids were used as negative and positive controls, respectively. Primer sequences for detection of episomal plasmids and pluripotency markers are listed in Supplementary Table S1.

### 2.5. Immunofluorescence Staining

iPSCs were cultured on 96-well black plates, fixed with 4% paraformaldehyde for 20 min, and then permeabilized with 0.1% Triton X-100 for 15 min and blocked with 2.5% BSA for 30 min. Cells were incubated overnight at 4 °C with primary antibodies (Supplementary Table S2), then incubated with secondary antibodies for 60 min. Nuclei were stained with Hoechst 33342 and imaging was done using an Olympus BX51 microscope. 

### 2.6. Germ Layer Differentiation

iPSCs were dissociated using 0.5 mM EDTA and transferred to ultra-low attachment culture dishes in E8 medium (without TGF-β1 and FGF2) for embryoid body (EB) formation. After 2 days, EBs were transferred to Matrigel-coated dishes. The medium was changed to a differentiation medium: DMEM/F12 containing 20% FBS, L-glutamine, and non-essential amino acids for mesoderm and endoderm induction; or DMEM/F12 containing 50% neurobasal medium, B27, N2, and L-glutamine for ectoderm induction. After 14 days, the cells were fixed and analyzed by immunofluorescence staining with antibodies against TUJ1, SMA, and AFP. For the directed differentiation of endoderm, cells were treated with RPMI supplemented with 2% FBS, 100 ng/mL activin A (R&D systems, Minneapolis, MN, USA) and 5 µM CHIR 99021 (LC laboratories, Woburn, MA, USA) for the first day, followed by the same media without CHIR99021 for 2 days. For mesoderm, cells were treated with advanced RPMI (Thermo Scientific) supplemented with Glutamax (Thermo Scientific) and 5 µM CHIR 99021 for 2 days. For ectoderm, cells were treated with DMEM (Thermo Scientific) supplemented with 1 µM dorsomorphin (Merck), 10 µM SB431542 (Merck), and 1 µM PD0325901 (Tocris, Bristol, UK) for 8 days. Germ layer-specific primers listed in Supplementary Table S1 were used to determine the gene expressions by ΔΔCt method.

### 2.7. Kidney Differentiation and Cyst Induction

MUi027-A iPSCs were differentiated into kidney cell lineage as previously described with modifications [13]. Briefly, iPSCs were grown until semi-confluency in E8 media supplemented with 1 × penicillin/streptomycin. Cells were then detached by incubating in 0.5 mM EDTA at 37 °C for 5–7 min. The cell suspension was then prepared in E8 medium as 10,000 cells/ cm^2^ and seeded on 24-well plates precoated with Matrigel (0.05 mg/well). On the next day, media was changed to E8 supplemented with Matrigel (2% per well) and grown for 2 more days. On day 4, medium was changed to ARPMI medium supplemented with 1 × B27 and 2 µM CHIR 99021 for 36 h. From day 6 onwards, the media was changed to ARPMI with 1 × B27 every 2 days. For cyst induction, kidney organoids (25 days or older) were treated with blebbistatin (12.5 µM) for 9 days or forskolin (30 µM) for 3 days. The kidney organoid development and cyst formation were observed by light microscope or immunostaining with kidney markers. 

### 2.8. Mycoplasma Detection

Genomic DNA was extracted from iPSCs as previously described and mycoplasma detection was analyzed using mycoplasma detection set (Promokine) following the manufacturer’s instructions. 2 µL of extracted DNA or a negative control (DNA elution buffer) was added to the test reactions and PCR was run using the following program: initial denaturation at 95 °C for 2 min, followed by 94 °C for 30 s, 55 °C for 30 s, 72 °C for 30 s, in a total of 40 cycles. Eight µL of each PCR reaction was loaded onto a 1.5% agarose gel prepared with RedSafe™ Nucleic Acid Staining Solution (IntronBio, Seongnam-si, Korea) and run for 25 min at 100 V.

## 3. Results

### 3.1. Generation of MUi027-A hiPSC Line

**Figure 1:**
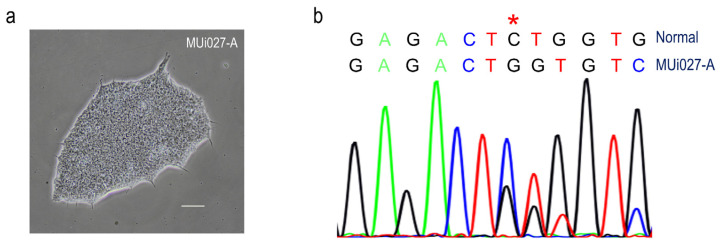
Generation of iPSC lines from ADPKD patient. (**a**) Representative bright field photograph of an iPS colony obtained from fibroblasts of a patient carrying *PKD1* mutation. (**b**) DNA sequencing result from exon 21 of the *PKD1* gene, showing 7946_7947delCT mutation. The asterisk symbol (*) indicates the position of frameshift mutation.

A human iPSC line (MUi027-A) was successfully reprogrammed into iPSCs by transfection with non-integrating episomal vectors. Fibroblasts were obtained from a skin biopsy of an ADPKD patient carrying the PKD1 mutation (c.7946_7947delCT), and episomal vectors encoding transcription factors were electroporated into the fibroblasts. Four weeks after electroporation, cells displayed an iPSC-like morphology (Figure 1a). DNA sequencing also confirmed that the MUi027-A iPSCs harbored the *PKD1* frameshift mutation (c.7946_7947delCT) at exon 21 (Figure 1b).

### 3.2. Characterization of MUi027-A hiPSC Line

**Figure 2:**
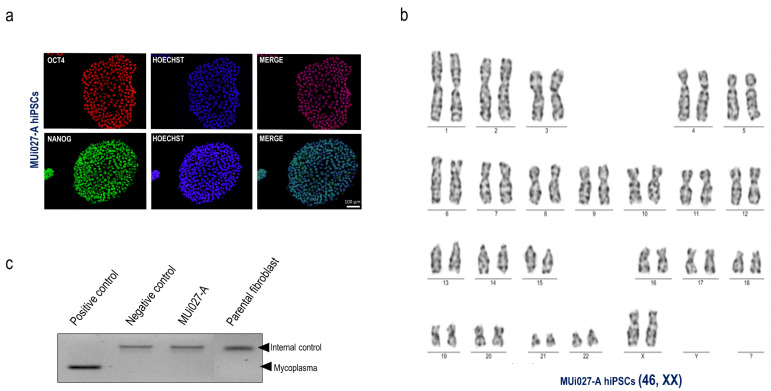
Characterization of human iPSC line MUi027-A. (**a**) Immunostaining of MUi027-A hiPSCs with OCT-4, and NANOG. Scale bars: 100 μm. (**b**) Karyotyping of MUi027-A iPSCs. (**c**) PCR gel electrophoresis results for detection of mycoplasma contamination in MUi027-A iPSCs, including parental fibroblasts.

MUi027-A hiPSCs were assessed for expression of stem cell markers after successful maintenance over 20 passages. Immunocytochemistry analysis showed robust expression of pluripotency markers OCT4 and NANOG (Figure 2a). A karyotyping analysis by G-banding revealed no chromosomal abnormality (Figure 2b). PCR amplification of mycoplasma-specific DNA sequences demonstrated that the MUi027-A cells were mycoplasma-free (Figure 2c). Additionally, STR analysis at 15 loci revealed an identical match between the reprogrammed iPSC line MUi027-A and the paternal fibroblasts (data not shown to protect patient identification).

### 3.3. Pluripotency Property Assessment of MUi027-A hiPSCs

**Figure 3:**
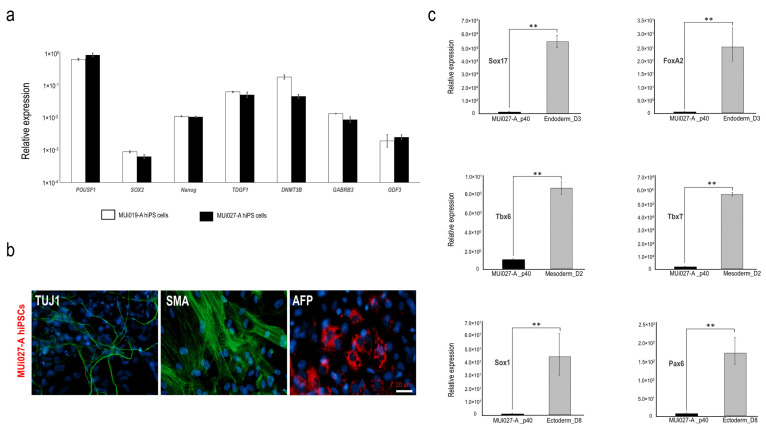
Pluripotency properties of MUi027-A iPSCs. (**a**) Real-time PCR of pluripotency genes in MUi027-A iPSCs. Expression levels of pluripotency genes are compared to those in previously published human iPSC line (MUi019-A). (**b**) Immunostaining of MUi027-A hiPSCs with TUJ1, SMA, and AFP. Expression levels are normalized to GAPDH. (**c**) Differentiation of MUi027-A hiPSCs into the three germ layers. Graphs depict the relative expression level of germ layer differentiation using real-time PCR for *Sox17* and *FoxA2* (Endoderm), *Tbx6* and *TbxT* (Mesoderm), and *Sox1* and *Pax6* (Ectoderm). Expression levels are compared to those in undifferentiated iPSCs. Relative expression values are normalized to GAPDH. Data represents mean ± SEM (*n* = 3), ** *p* < 0.01.

Quantitative (RT-PCR) analyses showed that MUi027-A hiPSCs actively expressed endogenous pluripotent factors similar to a previously published human iPSC line MUi019-A (Figure 3a). In vitro differentiation followed by immunofluorescence staining analysis with the ectodermal marker, β-tubulin (TUJ1); the mesodermal marker, smooth muscle actin (SMA); and the endodermal marker, α-feto protein (AFP), demonstrated the potential of differentiation into the three germ layers (Figure 3b). Furthermore, RT-PCR analyses also confirmed the expression of ectodermal (*Sox1*, *Pax6*), mesodermal (*Tbx6*, *TbxT*), and endodermal (*Sox17*, *FoxA2*) genes, respectively (Figure 3c). 

### 3.4. MUi027-A hiPSCs Developed into Kidney Organoids with Characteristics of PKD

**Figure 4:**
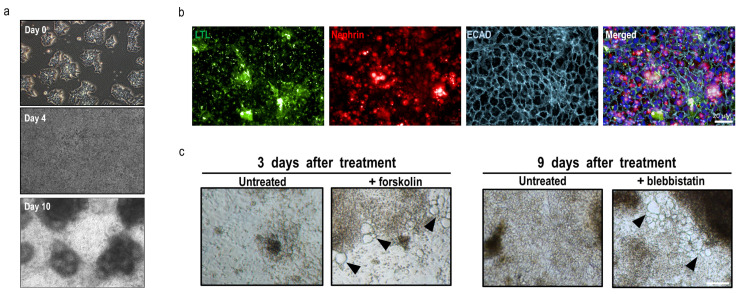
Directed differentiation of kidney organoids and cyst induction from MUi027-A hiPSCs. (**a**) Differentiation of MUi027-A into tubular organoids via CHIR treatment. Tubule-like structures were observed after day 8–10 post differentiation (**b**) Immunocytochemistry of organoid tubules stained with kidney markers (lotus tetragonolobus lectin; LTL, nephrin, and E-cadherin; ECAD). (**c**) Phase-contrast micrographs showing the effect of treatment with 30 µM forskolin for 3 days (*left*) or 12.5 µM blebbistatin for 9 days (*right*).

Kidney cysts are one of the striking features of ADPKD patients [2]. Using a previously published directed differentiation protocol involving the inhibition of glycogen synthase kinase-3β (GSK3β) [20], MUi027-A hiPSCs were successfully differentiated into tubular organoids (Figure 4a). These tubular structures displayed expression of various kidney markers such as Lotus tetragonolobus lectin (a proximal tubule marker), E-cadherin (a distal tubule marker), and nephrin (a marker of glomerulus) (Figure 4b). Previously, cyst-like swellings on organoid tubules were reported upon the addition of forskolin or blebbistatin [21,22]. Similarly, the addition of either forskolin (3 days) or blebbistatin (9 days) resulted in the development of multiple cyst-like swellings in MUi027-A-derived organoids (Figure 4c). The addition of either forskolin or blebbistatin to organoids developed from the normal hiPSC line (MUi019-A) did not result in the formation of cystic swellings. Furthermore, reduced cyst formation was observed when forskolin or blebbistatin was added to the organoids developed from the hiPSC line with a different mutation MUi026-A: *PKD1* gene (exon 15 with c.5878C>T) (Supplementary Figure S1), an hiPSC line we previously reported [23].

## 4. Discussion

Autosomal dominant polycystic kidney disease (ADPKD) is considered the most common inherited single-gene disorder which results in end-stage renal disease [24]. Following diagnosis, current therapeutic guidelines for PKD treatment aim for palliative care to reduce the complications of the PKD, without addressing the underlying pathogenesis [25]. However, only a few patient-derived iPSC lines with the ADPKD genotype are available in the public cell banks. Here, we reported a novel human iPSC line MUi027-A from fibroblasts of a patient with ADPKD genotype and demonstrated its potential to be used as a model for studying PKD disease. 

In the present experiments, an MUi027-A hiPSC line was generated by a non-integrating episomal system which does not require genome integration for the delivery of reprogramming factors [26,27]. DNA fingerprinting of reprogrammed iPSCs maintained under culture conditions indicated that the MUi027-A hiPSCs were derived from the donor material and the presence of *PKD1* frameshift mutations at the target loci was confirmed via DNA sequencing. Moreover, MUi027-A hiPSCs expressed pluripotency markers and maintained normal karyotype at higher passages (*p* > 15). When germ layer differentiation potential of MUi027-A was compared to the previously characterized human iPSC line MUi019-A [28], no significant differences were detected. Mutations within *PKD1* gene are reportedly associated with earlier onset of end-stage renal disease when compared to those with *PKD2* mutations [9]. Even among *PKD1* mutants, truncating mutations were associated with more severe renal disease than non-truncating mutations. Previously, we reported the generation of a hiPSC line (MUi026-A) from an ADPKD patient with a nonsense mutation (c.5878C > T) in the *PKD1* gene [3]. Since these iPSC lines harbor different *PKD1* mutations as a genotype background, we directly compared their potential to be differentiated into kidney organoids. Based on the previously published protocols for directed kidney differentiation [21,22], we obtained tubular structures with the characteristics of proximal tubular segments as detected by Lotus tetragonolobus lectin (LTL), including the expression of intercellular junction marker E-cadherin. Furthermore, the obtained tubular organoids also expressed nephrin, which is the surface protein of glomerular podocyte-like cells [29]. Tubular-like structures were visible around day 10 after induction and these clusters differentiated into tubular organoids around week 3. Similar timings were reported following the differentiation of human iPSC into tubular structures and subsequent development of kidney organoids [22]. We also found that under the conditions tested, MUi027-A and MUi026-A cell lines were able to differentiate into kidney organoids. However, different *PKD1* mutations may constitute multiple forms of phenotypic manifestations during disease development. Next, we explored the effect of stimulation with either forskolin or blebbistatin on cyst development in hiPSC lines with normal or ADPKD genotypes. Blebbistatin is a non-muscle myosin II ATPase inhibitor that has been shown to increase cyst formation significantly. Polycystins, which generally act as regulatory elements of actomyosin activation within the tubular epithelium to support normal tissue structure, could be involved in this phenomenon [22]. Forskolin is a cell-permeable compound which can induce cyst formation by activating adenylate cyclase, which converts adenosine triphosphate (ATP) into adenosine cyclic monophosphate (cAMP) and stimulates cellular proliferation and transepithelial fluid secretion via RAS/RAF/ERK and Wnt pathways [30]. Upon addition of either forskolin or blebbistatin, significant cyst formation was observed in tubular organoids from the MUi027-A hiPSC line where unstimulated organoids did not spontaneously form cysts, in line with previous reports [21,22]. However, only a few cysts were observed in organoids originating from the hiPSC line MUi026-A under similar conditions, whereas no visible cyst formation was observed in tubular organoids originating from the normal hiPSC line MUi019-A. Since MUi026-A has a *PKD1* point mutation (c.5878C > T) in contrast to the truncating mutation in MUi027-A (c.7946_7947delCT), the observed differences in the cyst formation could therefore represent the genotypic influences on the correlating phenotype development. Furthermore, these results are in line with the previously published report indicating that the median age at onset of end-stage renal failure was 55 years for carriers of a truncating mutation and 67 years for carriers of a non-truncating mutation [31]. This suggests that the mutation in *PKD1* gene is likely to be responsible for the development of cysts and the underlying physiological differences exist in the mechanisms of cyst formation between different *PKD1* mutations.

Patient-derived stem cell lines are capable of self-renewal and can be readily differentiated into all cell lineages and are essential to the study of multisystemic pathophysiology. Furthermore, they are a valuable drug screening platform for the development of novel therapies. In the current work, we demonstrated that the MUi027-A hiPSC line harbors the *PKD1* genotype and maintains the ability to be differentiated into somatic lineages, including kidney organoids. We also demonstrated that kidney organoids derived from MUi027-A could be readily induced to form cysts following forskolin/blebbistatin treatment. Our study, however, has important limitations as findings are based on the iPSC-derived kidney organoids which may not accurately recapitulate in vivo environment, as tubular organoids were developed during a few weeks in culture in contrast to the long-term development of ADPKD in clinical settings. Further, our kidney organoids lacked in vivo-like functional vasculature. Nevertheless, in their current form, the tubular organoids were shown to display specific kidney markers. Furthermore, organoids generated from normal hiPSCs responded differently to external stimuli when compared to those generated from hiPSCs with the *PKD1* mutation genotype, highlighting the important role of *PKD1* in ADPKD development. Altogether, the iPSC model of ADPKD will be useful in elucidating the genetic basis of the disease and the MUi027-A established in this study will provide an extra tool to advance the understanding of disease mechanisms, including the role of PKD1 mutations in the development of ADPKD.

## 5. Conclusions

Here, we report the successful establishment of a patient-derived iPSC line MUi027-A harboring a *PKD1* frameshift mutation (c.7946_7947delCT). We also found that iPSC-derived kidney organoids with different *PKD1* mutation types exhibited differences in cyst formation (a hallmark of ADPKD) in vitro, and thus support the notion that allelic influences of the *PKD1* mutation must be characterized in detail to fully understand the role of such mutations on disease development. Furthermore, accounting for the genetic background is critical to gain a deeper understanding of the mechanism of ADPKD and to explain the variations in disease development in correlation with genetic modifiers of disease severity.

## Data Availability

Not applicable.

## Supplementary Materials

The following supporting information can be downloaded at: https://www.mdpi.com/article/10.3390/jpm12050766/s1, Figure S1: Cyst induction in iPSC lines; Table S1: List of primers; Table S2. List of antibodies.
