# Supplementary material for: Establishment and Characterization of MUi027-A: A Novel Patient-Derived Cell Line of Polycystic Kidney Disease with PKD1 Mutation
Source: J Pers Med. 2022 May 9;12(5):766. doi: 10.3390/jpm12050766 (PMC9145395; doi:10.3390/jpm12050766)
Supplement: Supplementary file 1 [file jpm-12-00766-s001.zip › Supplementary Figure S1.pdf]

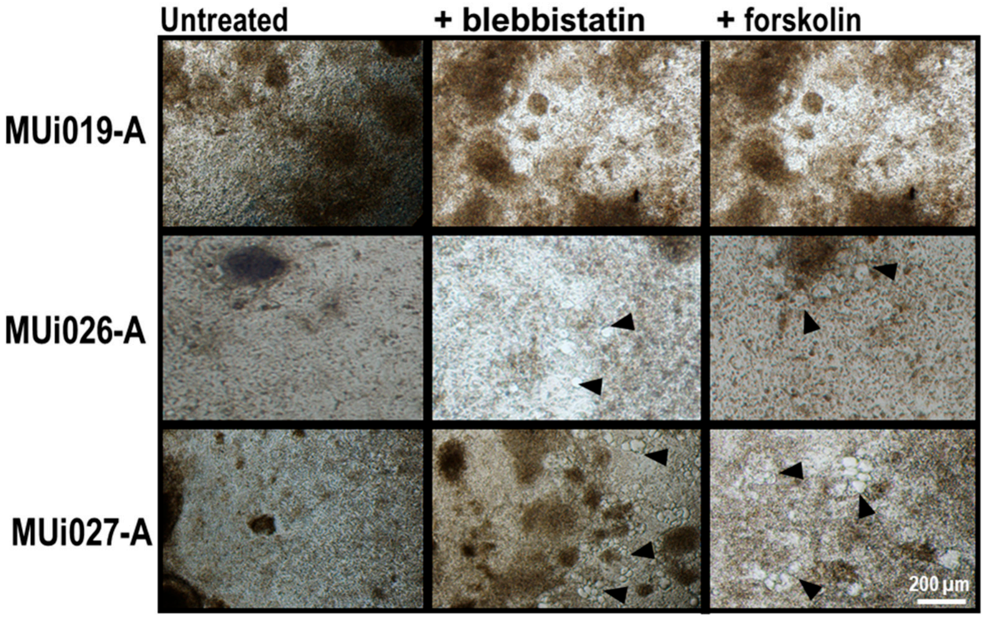

Figure S1. Cyst induction in hiPSC lines. Induction of cyst formation from hiPSC cell line-derived tubular organoids treated with either forskolin or blebbistatin. Tubular organoids were observed after day 20 of the differentiation and treated with either forskolin (3 days) or blebbistatin (9 days). The extent of cyst formation was analyzed by phase contrast microscopy.
