# Supplementary material for: Establishment and Characterization of MUi027-A: A Novel Patient-Derived Cell Line of Polycystic Kidney Disease with PKD1 Mutation
Source: J Pers Med. 2022 May 9;12(5):766. doi: 10.3390/jpm12050766 (PMC9145395; doi:10.3390/jpm12050766)
Supplement: Supplementary file 1 [file jpm-12-00766-s001.zip › Supplementary Table S1.pdf]

Table S1. List of primers

	Target	Forward/Reverse primer (5′-3′)
EBNA1	EBNA1	F: ATCGTCAAAGCTGCACACAG /R: CCCAGGAGTCCCAGTAGTCA
Pluripotency Markers (qPCR)	DNMT3B	HS00171876_m1
	OCT4	HS03005111_g1
	GDF3	HS00220998_m1
	NANOG	HS02387400_g1
	GABRB3	HS00241459_m1
	SOX2	HS00602736_s1
	TDGF1	HS02339497_g1
	GAPDH	HS99999905_m1
Pluripotency Markers (qPCR)	*Pax6*	F: TGGGCAGGTATTACGAGACTG /R: ACTCCCGCTTATACTGGGCTA
	Sox17	F: GAGCCAAGGGCGAGTCCCGTA /R: CCTTCCACGACTTGCCCAGCAT
	FoxA2	F: GGAGCAGCTACTATGCAGAGC /R: CGTGTTCATGCCGTTCATCC
	TBX6	F: CATCCACGAGAATTGTACCCG /R: AGCAATCCAGTTTAGGGGTGT
	TBXT	F: ACCCAGTTCATAGCGGTGAC /R: CCATTGGGAGTACCCAGGTT
	SOX1	F: CAGTACAGCCCCATCTCCAAC /R: GCGGGCAAGTACATGCTGA
