# Supplementary material for: Establishment and Characterization of MUi027-A: A Novel Patient-Derived Cell Line of Polycystic Kidney Disease with PKD1 Mutation
Source: J Pers Med. 2022 May 9;12(5):766. doi: 10.3390/jpm12050766 (PMC9145395; doi:10.3390/jpm12050766)
Supplement: Supplementary file 1 [file jpm-12-00766-s001.zip › Supplementery Table S2.pdf]

Table S2. List of antibodies.

	Antibody	Dilution	Company Cat # and RRID
Pluripotency markers	Mouse anti-OCT4<br><br>Rabbit anti-NANOG	1:100<br><br>1:100	Santa Cruz Cat# SC-5279,<br>RRID: AB_628051<br>Santa Cruz Cat# SC-33759,<br>RRID: AB_2150401
Differentiation Markers	Mouse anti-β3 tubulin (ectodermal)<br>Mouse anti-SMA (mesodermal)<br>Goat anti-AFP (endodermal)<br>Mouse anti-E Cadherin<br><br>Lotus Tetragonolobus Lectin (LTL), FITC-conjucated<br>Anti-nephrin Antibody	1:100<br><br>1:100<br><br>1:100<br><br>1:250<br><br>1:250<br><br><br>1:100	Santa Cruz Cat# sc-51670,<br>RRID: AB_630408<br>Santa Cruz Cat# sc-53142,<br>RRID: AB_2273670<br>Santa Cruz Cat# sc-8108,<br>RRID: AB_633815<br>Abcam Cat#ab1416,<br>RRID: AB_300946<br>Vector Laboratories Cat# FL-1321-2<br>RRID: AB_ 2336559<br><br>R&D systems Cat# AF-4269
Secondary antibodies	Alexa Flour 594 goat anti-mouse IgG (H+L)<br>Alexa Flour 488 goat anti-rabbit IgG (H+L)<br>Alexa Flour 488 goat anti-mouse IgG (H+L)<br>Alexa Flour 594 donkey anti-Goat IgG (H+L)<br>Alexa Flour 647 donkey<br>Anti-Sheep IgG (H&L)	1:1000<br><br>1:1000<br><br>1:1000<br><br>1:1000<br><br>1:1000	Thermo Fisher Scientific Cat# A11005,<br>RRID: AB_2534073<br>Thermo Fisher Scientific Cat# A11008,<br>RRID: AB_143165<br>Thermo Fisher Scientific Cat#A11001,<br>RRID: AB_2534069<br>Thermo Fisher Scientific Cat# A11058,<br>RRID: AB_2534105<br>Thermo Fisher Scientific Cat# A21448,<br>RRID: AB_10374882
